# High Performance Eight-Port Dual-Band MIMO Antenna System for 5G Devices

**DOI:** 10.3390/mi13060959

**Published:** 2022-06-17

**Authors:** Saad Hassan Kiani, Muhammad Abbas Khan, Umair Rafique, Mohamed Marey, Abdullah G. Alharbi, Hala Mostafa, Muhammad Amir Khan, Syed Muzahir Abbas

**Affiliations:** 1 Department of Electrical Engineering, IIC University of Technology, Phnom Penh 121206, Cambodia; 2 Smart Systems Engineering Laboratory, College of Engineering, Prince Sultan University, Riyadh 11586, Saudi Arabia; 3 Department of Electrical Engineering, Balochistan University of Information Technology, Engineering and Management Sciences, Quetta 1800, Pakistan; muhammad.abbas@buitms.edu.pk; 4 Department of Information Engineering, Electronics and Telecommunications, Sapienza University of Rome, 00184 Rome, Italy; umair.rafique@uniroma1.it; 5 Department of Electrical Engineering, Faculty of Engineering, Jouf University, Sakaka 42421, Saudi Arabia; a.g.alharbi@ieee.org; 6 Department of Information Technology, College of Computer and Information Sciences, Princess Nourah bint Abdulrahman University, Riyadh 11671, Saudi Arabia; hfmostafa@pnu.edu.sa; 7 Department of Computer Science, COMSATS University, Abbottabad Campus, Abbottabad 22020, Pakistan; amirkhan@cuiatd.edu.pk; 8 Faculty of Science and Engineering, School of Engineering, Macquarie University, Sydney, NSW 2109, Australia; syed.abbas@mq.edu.au

**Keywords:** MIMO, 5G, dual-band, parasitic element

## Abstract

This study provides an eight-component multiple-input multiple-output (MIMO) antenna architecture for fifth-generation (5G) mobile communication systems. The single antenna element is comprised of an L-shaped radiating component, an L-shaped parasitic element, and a ground plane with a rectangular slot. The main element with a slot-loaded ground plane helps to draw current from a coaxial feed from the other side of the board, while the parasitic element helps to elongate the current path and improve the impedance of the system. This enables the system to radiate at two different frequency ranges: 3.34–3.7 GHz and 4.67–5.08 GHz, with 360 MHz and 410 MHz bandwidths, respectively. For MIMO configuration, the radiating elements are designed on either side of a 0.8 mm thick FR-4 substrate, allowing space to accommodate a battery, radio frequency (RF) systems and subsystems, and camera and sensor modules. The corner and the middle elements are arranged in such a manner so that they can provide spatial and pattern diversity. Furthermore, at least 12 dB of isolation is established between any two radiating elements. Various MIMO performance parameters were evaluated, e.g., mean effective gain (MEG), channel capacity (CC), envelope correlation coefficient (ECC), realized gain, far-field characteristics, and efficiency. Single- and double-hand mode evaluations were performed to further demonstrate the capability of the proposed MIMO antenna. A prototype of the proposed MIMO antenna was manufactured and assessed to verify the simulated data. The measured and simulated results were found to be in good agreement. On the basis of its performance characteristics, the designed MIMO system could be used in 5G communication systems.

## 1. Introduction

Communication systems using fifth-generation (5G) infrastructure are expected to provide faster data speeds, better connections, and reduced latency than rivals, including long-term evolution (LTE) networks [1,2,3,4]. The International Telecommunication Union (ITU) has authorized the mid-band of 3.5 GHz, 4.8 GHz, and 5.8 GHz for 5G communication systems. This is also referred to as the sub-6 GHz spectrum, whose main purpose is to maximize coverage while reducing propagation losses. Additionally, a sub-6 GHz network needs at least six radiating antenna elements for increased ergodic channel capacity (CC).

Several multiple-input multiple-output (MIMO) antennas for use in mobile phone applications operating in the sub-6 GHz spectrum have been discussed in the literature. In [5], a 10-element MIMO configuration was developed for mobile services. The single antenna of the MIMO arrangement was comprised of a T-resonator fed by an L-shaped microstrip line. Two MIMO elements were positioned on the upper and lower sides of the printed circuit board, and the remaining six elements were located on the other sides of the board. The array was formed to resonate in two frequency ranges, 3.5 GHz and 5.5 GHz. More than 10 dB of isolation between the antenna elements was observed for the 3.5 GHz frequency band and more than 15 dB of isolation was noted for the 5.5 GHz frequency band. For the 3.5 GHz frequency, the authors of [6] designed an eight-element MIMO configuration. A fork-shaped radiating element with an L-shaped feeding arrangement was developed. Although the design suffers from low antenna efficiency, this structure provides greater than 7.5 dB of isolation between antenna components. An eight-port MIMO antenna with broadband characteristics was designed in [7] for sub-6 GHz services. A 50 Ω microstrip feeding line, an open loop tuning stub, a slit on a metal frame, and a U-shaped slit on a ground plane made up the antenna of the MIMO array.

In [8], an eight-element monopole notched MIMO antenna in the 2.6–3.5 GHz frequency range was designed. Four antenna elements were mounted on the phone’s metal chassis and fed by an L-shaped feeding line. The remaining four antenna elements were mounted on the mobile phone’s top and bottom planes. In the operating bands, the MIMO configuration provided adequate reflection and isolation performance. In [9], 3.5 GHz MIMO antenna arrays for 5G cellphone usage were presented. Arrays were constructed by two distinct methods: an L-shaped coupled-fed array and a U-shaped loop array, which were both attached to the smartphone’s metal frame. The researchers employed an inverted-I gap and a neutralization line between the antenna components to achieve a 15 dB improvement in isolation. In [10], a sub-6 GHz cellular MIMO loop antenna array was developed. The antenna components and ground plane were both placed on the same face of the PCB. The band of interest was served by a loop of eight antenna components organized in such a way as to offer polarization and pattern diversity. Enhanced bandwidth (3.2–4 GHz) and isolation (>15 dB) were attained via applying an arrow-shaped microstrip structure between antennas. A comparable MIMO setup was shown in [11]. Planar inverted-F antennas (PIFAs) were used to resonate in three unique frequency ranges. In [12], a dual-band six-element MIMO antenna was designed. A loop-shaped resonator was designed to achieve resonance in two different 5G bands with an isolation of 10 dB. Most of the designs reported in the literature either suffer from poor antenna efficiency or complex design structures.

The aim of this work is to design a MIMO antenna that must be simple, easy to manufacture, and easily combined with other communication units within 5G systems. In addition, it should be lightweight, low-cost, and efficient without compromising the performance of the system. Furthermore, 5G systems are data and capacity hungry; therefore, the system should have reasonable capacity, low mutual coupling, and pattern diversity. For this purpose, an eight-element MIMO antenna is designed for dual-band 5G applications. The radiating elements are placed along the length of the PCB. The single antenna element of the proposed MIMO system consists of an L-shaped radiation element backed by a rectangular slot-loaded ground plane and an L-shaped parasitic element. It resonates at two different FCC-allocated 5G spectrums, 3.5 GHz and 4.8 GHz, and offers bandwidths of 360 MHz and 410 MHz, respectively. Moreover, the isolation between antenna components is noted to be >12 dB, which tends to achieve a low envelope correlation coefficient (ECC), an acceptable mean effective gain (MEG), and high CC. Based on the preceding highlights, it can be said that the MIMO system could be used as a potential solution in sub-6 GHz 5G systems where simplicity, efficiency, high data rates, special diverse radiation characteristics, and minimal interference between adjacent radiating components are desired.

## 2. Antenna Design

**Figure 1:**
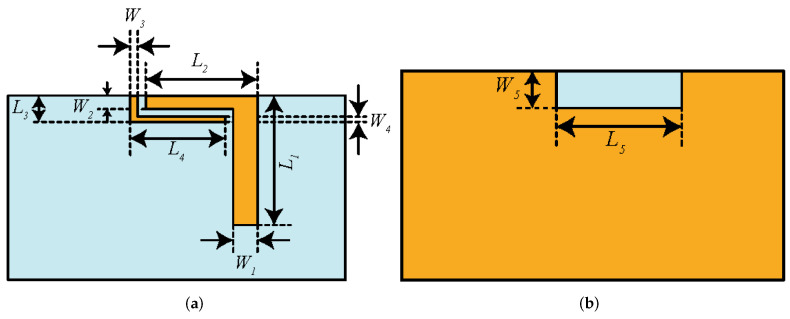
The proposed single antenna element: (**a**) front; (**b**) back.

Before presenting the MIMO configuration, there is a need to discuss the design and working principle of the single antenna component. Figure 1 shows a single antenna element design along with its dimensions. The antenna element is designed on a 0.8 mm thick FR-4 substrate having dielectric constant of 4.4. The reason for choosing the FR-4 substrate is its low cost and wide availability. Please note that this antenna element is etched on one face of the board, with copper backing on the other one. The other design parameters of a single antenna element are: $W_{1}$ = 3, $L_{1}$ = 17, $L_{2}$ = 11.5, $W_{2}$ = 1.5, $L_{3}$ = 2, $W_{3}$ = 1, $L_{4}$ = 12.5, $W_{4}$ = 0.5, $L_{5}$ = 14, and $W_{5}$ = 3 (all dimensions in mm).

**Figure 2:**
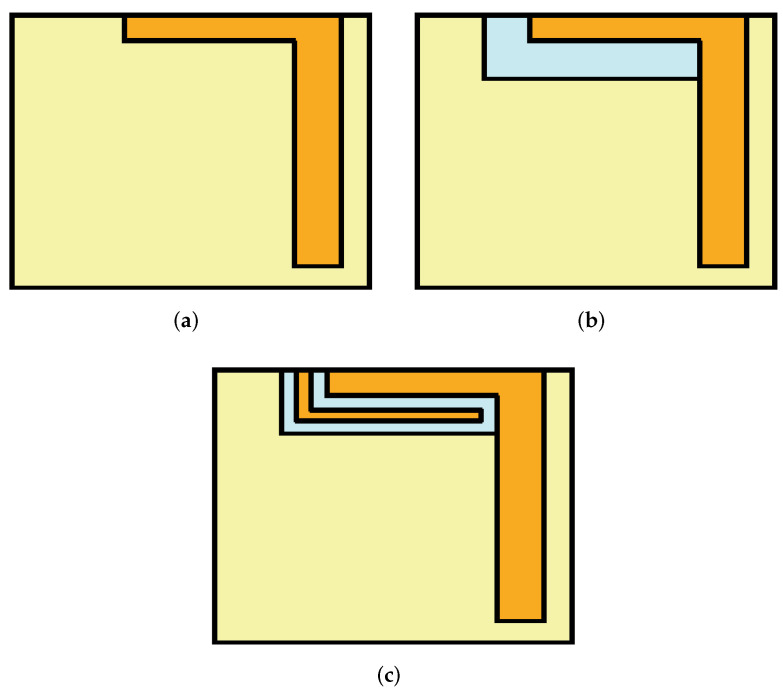
Design steps of single antenna element: (**a**) Step 1, (**b**) Step 2, and (**c**) Step 3. Orange, top metal; light blue, dielectric substrate; and light yellow, bottom metal.

The construction of the proposed radiating element is divided into three parts: the main element, the rectangular slot-loaded ground plane, and the parasitic element. The main element is composed of an L-shaped structure (see Figure 2a) whose resonant length (denoted as $L_{2}$) is ≈0.25$\lambda_{g}$ at 4.8 GHz, where $\lambda_{g}$ is the guided-wavelength and can be calculated using expression below [12].

(1) $$\lambda_{g}=\frac{\lambda_{0}}{\sqrt{\varepsilon_{reff}}}$$

where

(2) $$\lambda_{0}=\frac{c}{f_{r}}\;\mathrm{and}\;\varepsilon_{reff}=\frac{\varepsilon_{r}+1}{2}$$

where $\lambda_{0}$ is the free-space wavelength, *c* is the speed of light, $f_{r}$ denotes the resonant frequency, and $\varepsilon_{r}$ represents the relative permittivity of the dielectric substrate.

**Figure 3:**
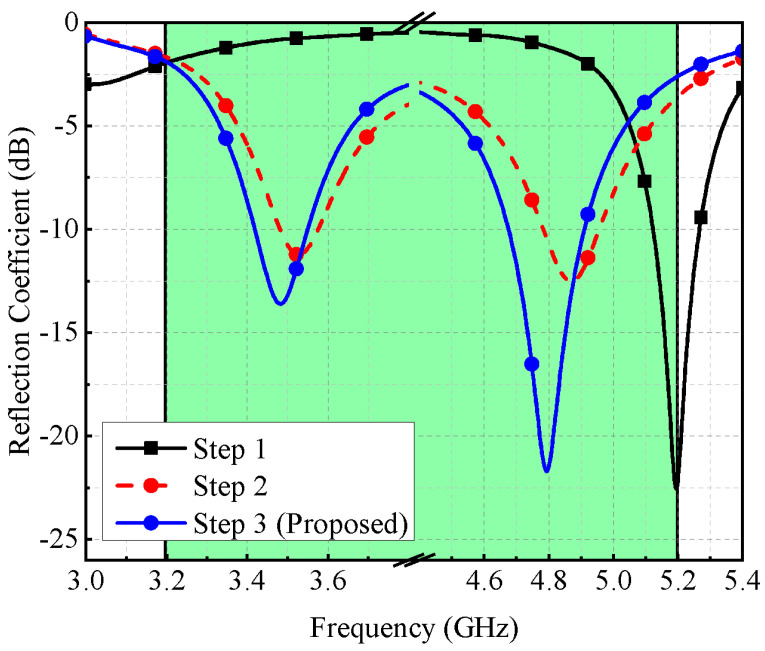
Reflection coefficient of different design stages.

**Figure 4:**
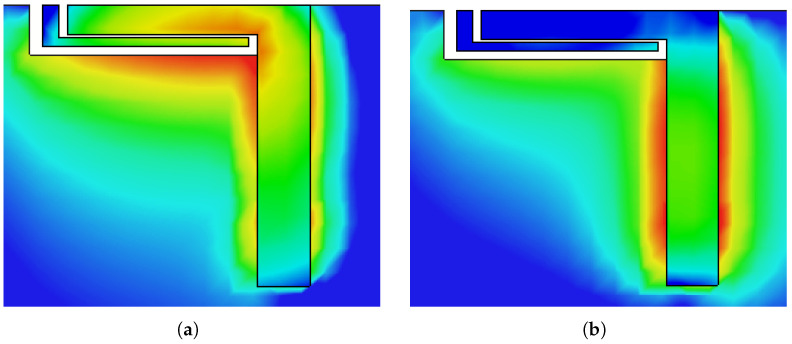
Surface current distribution of single antenna element at (**a**) 3.5 GHz and (**b**) 4.8 GHz.

As shown in Figure 3, the main element resonates at a single frequency band (around 5.2 GHz). When a slot is introduced in the ground plane (see Figure 2b), lower resonance is achieved around 3.5 GHz and higher resonance is also shifted to 4.9 GHz, thus generating a dual-band response (Step 2 of Figure 3). One can also say that the coupling between the radiation element and the slot-loaded ground plane is responsible for generating the dual-band response. The parasitic element is composed of an inverted L-shaped structure (see Figure 2c), which contributes to better impedance matching (see Figure 3, Step-3). From Figure 4, one can observe that once the excitation is turned on, the main element with a slot-loaded ground plane draws current from a coaxial feed placed on the opposite side of the board, which tends to achieve dual-band response. The parasitic element elongates the current path and improves the system’s impedance. From Figure 4a, one can observe that the L-shaped radiation element has a uniform current distribution over its surface, around the edges of the rectangular slot, and on the surface of the parasitic element, which tends to achieve dual-band response. On the other hand, from Figure 4b, it can be observed that the main radiating element is responsible for generating the 4.8 GHz frequency band, which can also be noted from the result of Figure 3 (Step 1).

**Figure 5:**
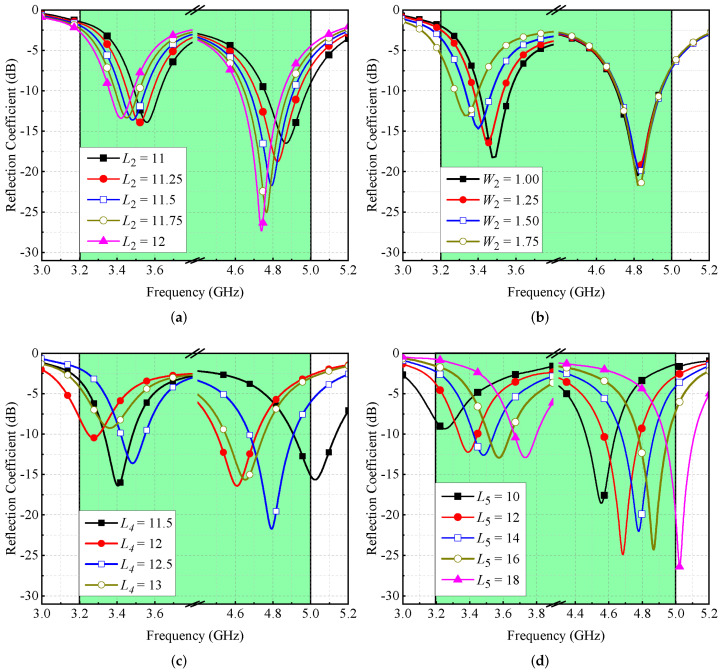
Effect of (**a**) $L_{2}$, (**b**) $W_{2}$, (**c**) $L_{4}$, and (**d**) $L_{5}$ on antenna’s reflection coefficient.

A parametric study is conducted to understand the behavior of a single antenna element. Four variables, $L_{2}$, $W_{2}$, $L_{4}$, and $L_{5}$ are investigated. Please note that $L_{2}$ and $W_{2}$ are the length and width of the main resonator, $L_{4}$ is the length of the parasitic element, and $L_{5}$ is the slot length in a ground plane. By adjusting the amounts of $L_{2}$, both resonances shift towards lower bands (see Figure 5a), and by varying $W_{2}$, there is a shift observed in the first resonance, as shown in Figure 5b. By varying the value of $L_{4}$, both resonances fluctuate in their frequency spectrum, as shown in Figure 5c. Similarly, by varying $L_{5}$, both resonances are affected (see Figure 5d). All these changes are expected because the structure is comprised of stubs. Therefore, the length and width of these stubs alter the resonant frequencies.

**Figure 6:**
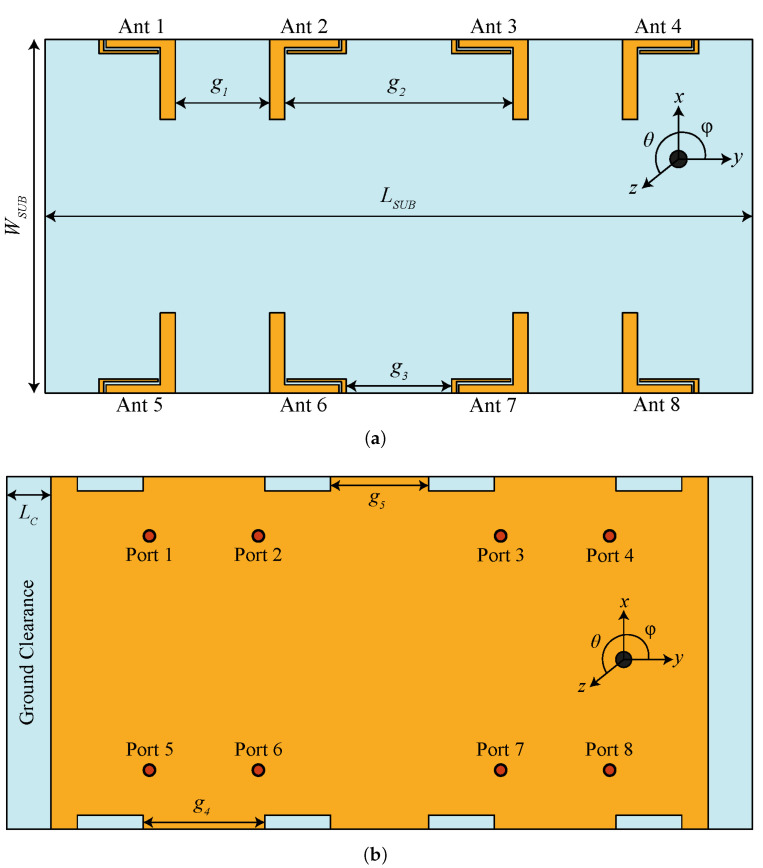
The proposed MIMO antenna system: (**a**) front; (**b**) back.

The MIMO system is modeled by incorporating a single antenna element into two linear sub-arrays placed on either side of the PCB in the vertical direction, as shown in Figure 6a. Each sub-array consists of four radiating elements. The corner and the center elements are arranged in different ways to improve isolation without using any decoupling structure or signal processing techniques (see Figure 6a). The ground plane consists of eight rectangular slots, and the antenna elements are fed using a 50 Ω coaxial connector, as shown in Figure 6b. The total dimensions of the board are 150 × 75 mm${}^{2}$, which is well-aligned with modern mobile phone dimensions. It should be noted that the overall area occupied by one antenna element equals 16.95 × 17 mm${}^{2}$, while the four elements occupy a space of about 128.7 × 17 mm${}^{2}$. It can be seen from Figure 6 that there is a lot of space available between antenna elements, which can be utilized to accommodate small parts and components of the mobile phone. The remaining design parameters are as follows: $L_{SUB}$ = 150, $W_{SUB}$ = 75, $g_{1}$ = 20, $g_{2}$ = 42.4, $g_{3}$ = 22.4, $g_{4}$ = 25.9, $g_{5}$ = 20.9, and $L_{C}$ = 5 (all dimensions in mm).

## 3. Fabrication and Measurement

**Figure 7:**
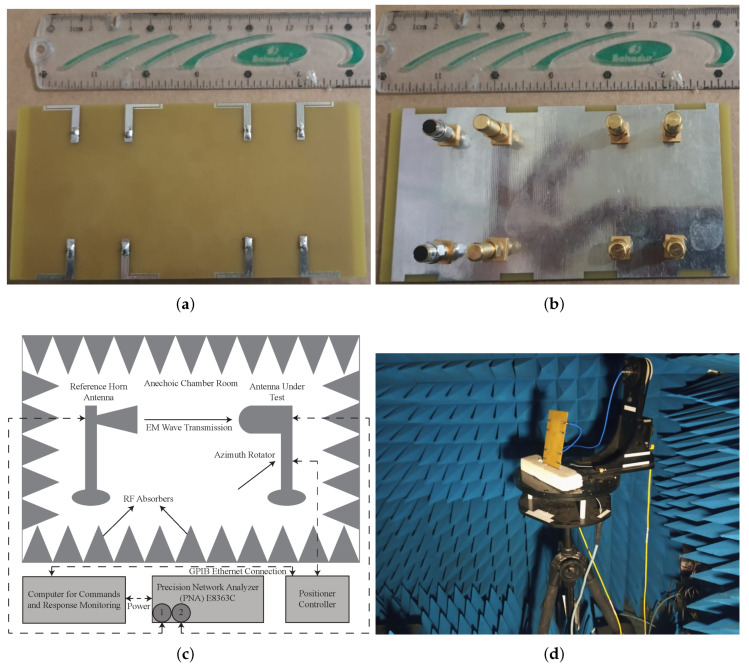
Fabricated prototype of MIMO antenna: (**a**) front; (**b**) back; (**c**,**d**) antenna far-field measurement setup.

Figure 7a,b show the manufactured prototype of the MIMO antenna. It is fabricated by utilizing an LPKF-D104 milling machine and measured using an Agilent Precision Network Analyzer (PNA-E8363C). The far-field characteristics are measured using an anechoic chamber, whose measurement setup is depicted in Figure 7c,d. From the figure, one can observe that the antenna under test (AUT) is placed horizontally on a turntable opposite to a horn antenna whose frequency range is 1–18 GHz, while the walls are covered with perfectly absorbing material.

**Figure 8:**
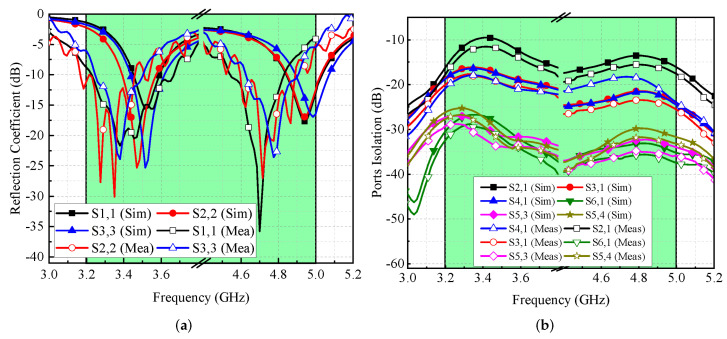
Simulated and measured scattering parameters of MIMO antenna (**a**) reflection coefficient and (**b**) isolation.

**Figure 9:**
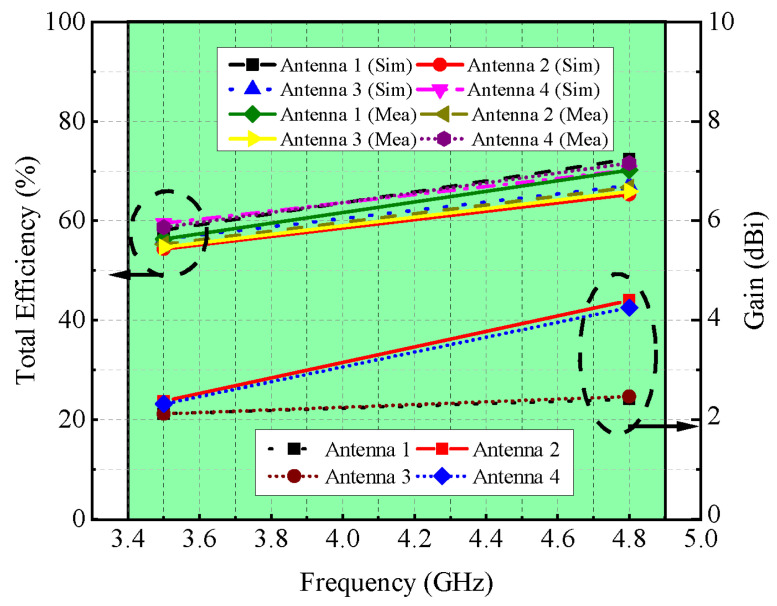
Gain and efficiency of MIMO antenna.

Figure 8a illustrates the modeled and actual reflection coefficients for port-1, port-2, and port-3. It is found that the MIMO antenna provides dual frequency response at the 3.5 GHz and 4.8 GHz frequency bands. The simulated −6 dB impedance bandwidths for both ranges are 360 MHz (3.34–3.70 GHz) and 410 MHz (4.67–5.08 GHz), respectively (see Figure 8a). On the other hand, the measured impedance bandwidths for both bands are noted to be 440 MHz (3.23–3.67 GHz) and 280 MHz (4.55–4.83 GHz), respectively, as shown in Figure 8a. The shifts in the frequency responses are caused by manufacturing tolerances, SMA connection losses, and measurement setup losses. The simulated and measured isolation performance of the proposed MIMO antenna is shown in Figure 8b. It is observed that the isolation performance lies in the acceptable range [13,14]. For adjacent antenna elements, isolation of ≥10 dB is noted for the 3.5 GHz frequency range and >12 dB for the 4.8 GHz frequency range, as shown in Figure 8b. The gain and overall efficiency of the suggested MIMO antenna are shown in Figure 9. The total efficiency for the 3.5 GHz and 4.8 GHz frequency bands ranged from 55% to 62% and 65% to 73%, respectively. Similarly, the gain for both the bands is well above 2 dBi, while the noted peak gain is around 4 dBi.

**Figure 10:**
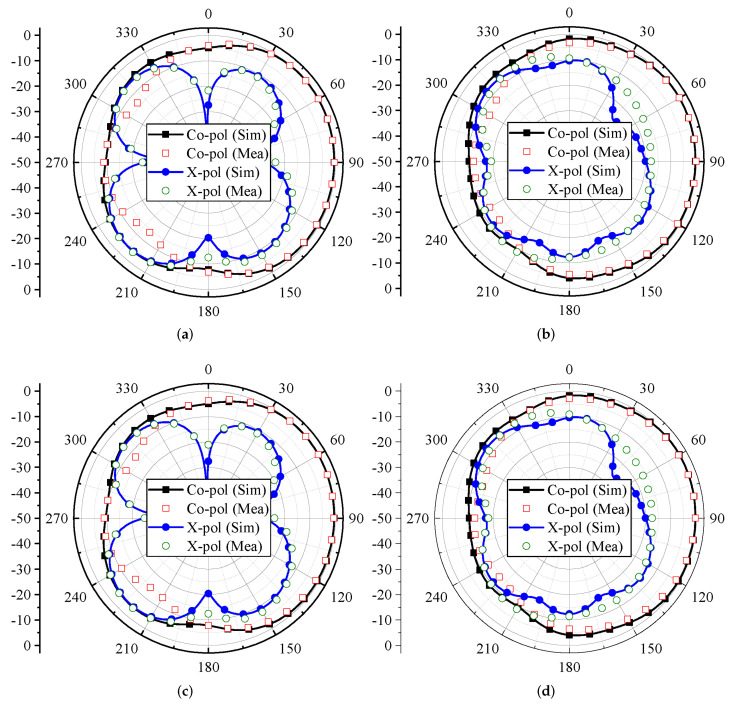
Modeled and actual radiation characteristics of MIMO antenna at ϕ = 0${}^{\circ }$ for Ant 1 at (**a**) 3.4 GHz and (**b**) 4.8 GHz; Ant 4 at (**c**) 3.4 GHz and (**d**) 4.8 GHz.

The far-field characteristics of the proposed MIMO antenna for the ϕ = 0${}^{\circ }$ plane are shown in Figure 10. The characteristics are evaluated for Ant 1 and Ant 4 in terms of co-polar (co-pol) and cross-polar (X-pol) components. For the 3.4 GHz and 4.8 GHz frequency bands, Ant 1 and Ant 4 offer directional radiation characteristics. The maximum power is directed towards θ = 90${}^{\circ }$, as shown in Figure 10. Furthermore, the X-pol level (see Figure 10) is well below −10 dB for both frequency bands. It can also be observed from Figure 10a,c that the X-pol pattern has a null at θ = 90${}^{\circ }$.

**Figure 11:**
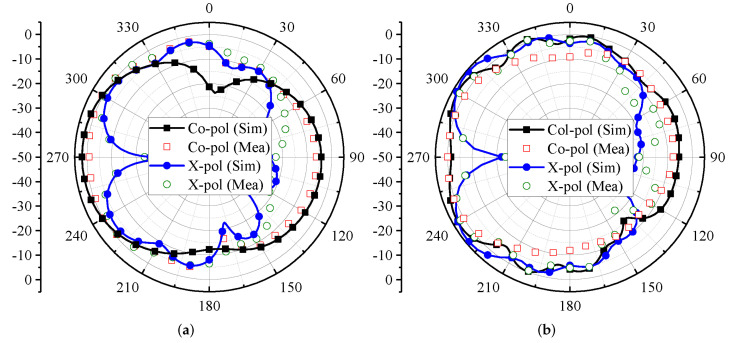

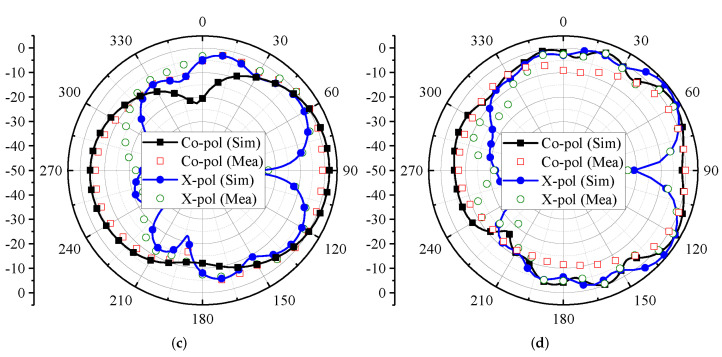
Modeled and actual radiation characteristics of MIMO antenna at ϕ = 90${}^{\circ }$ for Ant 1 at (**a**) 3.4 GHz and (**b**) 4.8 GHz; Ant 4 at (**c**) 3.4 GHz and (**d**) 4.8 GHz.

For the ϕ = 90${}^{\circ }$ plane, the radiation characteristics of Ant 1 and Ant 4 for the 3.5 GHz and 4.8 GHz frequency bands are shown in Figure 11. Both the antennas offer directional radiation characteristics for the 3.4 GHz and 4.8 frequency bands. In the case of Ant 1, the maximum power is directed towards θ = 270${}^{\circ }$ (see Figure 11a,b), while for Ant 4, the maximum power is directed towards θ = 90${}^{\circ }$, as shown in Figure 11c,d. This property also ensures that Ant 1 and Ant 4 offer pattern diversity for the ϕ = 90${}^{\circ }$ plane. Furthermore, by considering X-pol patterns, a null has been observed in the radiation direction. It can also be observed from Figure 11 that at some angles the level of X-pol patterns is high, which may be the effect of higher-order modes.

## 4. Diversity Performance Analysis

Next, the diversity performance analysis of the designed MIMO antenna system is thoroughly described. The performance is evaluated in terms of MEG, ECC, and CC.

MEG is an important characteristic of MIMO antenna systems that reflects the gain of the system within a multipath environment. It can be calculated by using the expression defined by [15]. The MEG of the proposed MIMO antenna is listed in Table 1. From the data in the table, one can observe that the value of MEG is less than 1 dB.

ECC illustrates how well radiating elements in an array are isolated [12,16]. It can be calculated by using the expression given in [12].

(3) $$ECC={\left|\frac{{\;\int \int \;}_{4\pi }S_{i}\left(\theta ,\phi \right).S_{j}^{*}\left(\theta ,\phi \right)d\Omega }{\sqrt{{\;\int \int \;}_{4\pi }S_{i}\left(\theta ,\phi \right).S_{i}^{*}\left(\theta ,\phi \right)d\Omega {\;\int \int \;}_{4\pi }S_{j}\left(\theta ,\phi \right).S_{j}^{*}\left(\theta ,\phi \right)d\Omega }}\right|}^{2}$$

where $S_{i}$ and $S_{j}$ represent far-field radiation characteristics of port *i* and port *j*, respectively, and dΩ is the solid angle.

**Figure 12:**
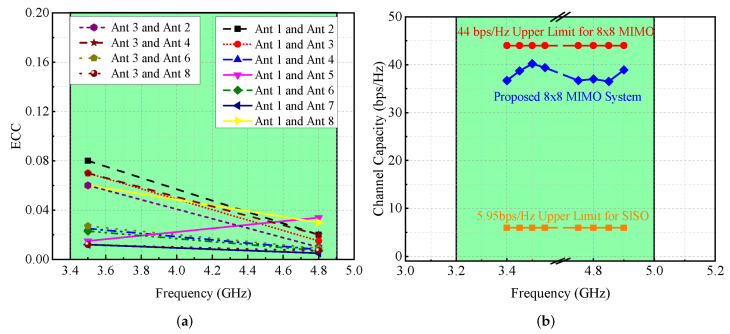
(**a**) ECC and (**b**) ergodic CC of MIMO antenna.

For the suggested MIMO antenna, the calculated ECC for both frequency bands, as shown in Figure 12a, is less than 0.1, which indicates that interaction between antenna components is minor.

Figure 12b depicts the proposed MIMO antenna system’s ergodic CC, which is in the 35–41 bps/Hz range. This value is close to the ideal eight-element CC, which is equal to 44 bps/Hz [17] (see Figure 12b). Mathematically, ergodic channel capacity can be calculated using the equation presented in [16]. Please note that the CC of the suggested antenna is computed by averaging ten thousand Rayleigh fading observations at an SNR of 20 dB [18].

## 5. User’s Effect on MIMO Antenna Performance

**Figure 13:**
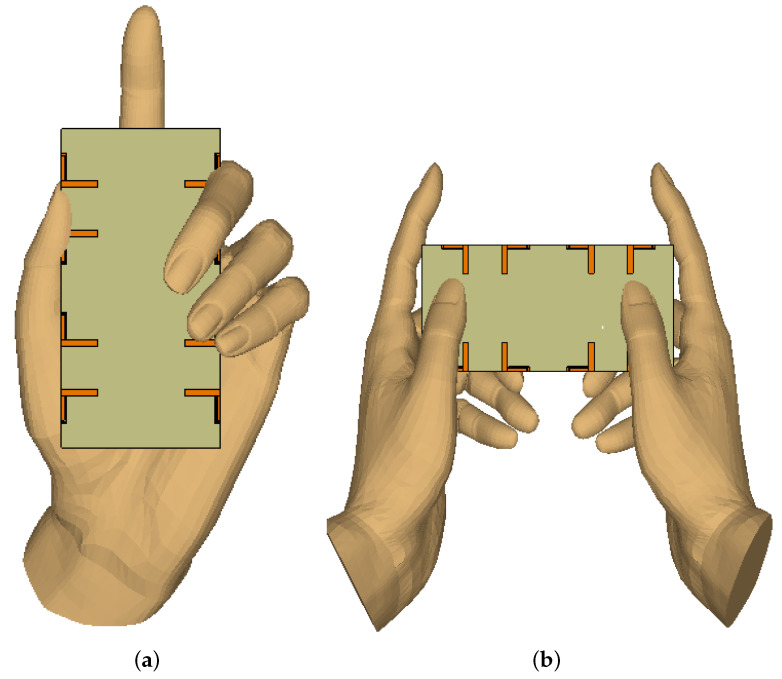
(**a**) Single-hand and (**b**) double-hand mode.

As mentioned earlier, this work aims to design a simple, compact, and easy-to-integrate antenna system that may be used as a modern mobile terminal. Therefore, it is necessary to investigate performance in different real-life scenarios, including single-hand mode (SHM) and dual-hand mode (DHM), as indicated in Figure 13. These studies are conducted to evaluate the impact of such cases on the performance and reliability of the system. Here, we considered many factors, for instance, antenna design and assembly, their distance from the palms of the hands, and so on. For the specified electric characteristics of a customer’s hand, it is assumed that the dielectric constant is 29 and effective conductivity is 0.8 S/m for both frequency bands. This investigation includes key performance characteristics, namely reflection coefficient, isolation, efficiency, and ECC.

**Figure 14:**
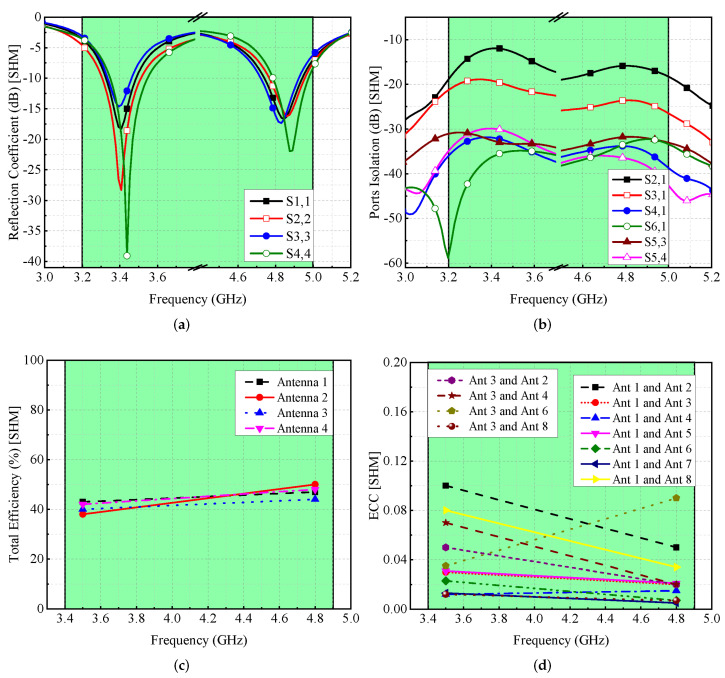
Effect of single-hand on MIMO antenna’s (**a**) reflection coefficient, (**b**) isolation, (**c**) efficiency, and (**d**) ECC.

**Figure 15:**
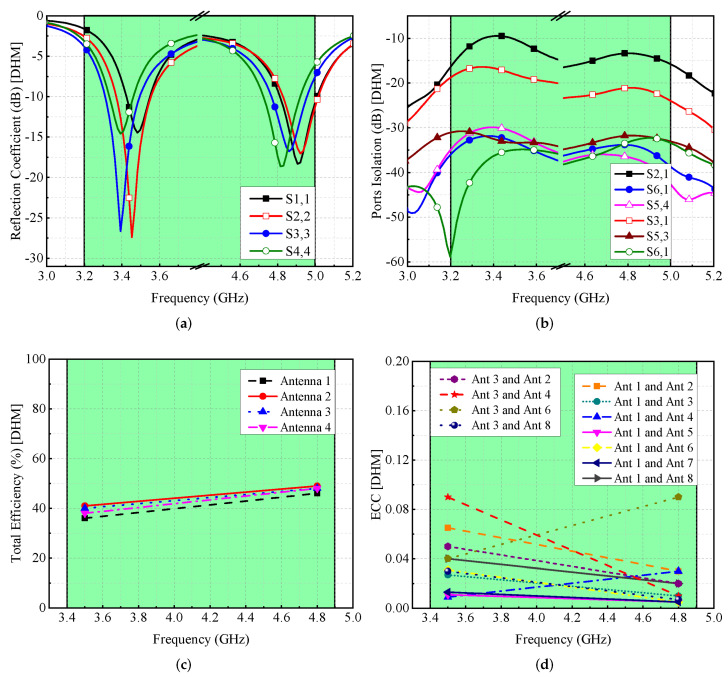
Effect of double-hand on MIMO antenna’s (**a**) reflection coefficient, (**b**) isolation, (**c**) efficiency, and (**d**) ECC.

For SHM, reflection coefficients and isolation performance are computed and presented in Figure 14. It is found that the resonances are shifted but still cover the desirable frequency bands (see Figure 14a). These shifts are due to the dielectric loading of the hand (fingers close to the antenna). Similarly, the isolation between any two antenna components is more than 12 dB, as indicated in Figure 14b. The total efficiency of the antenna elements ranges between 40–50% (see Figure 14c), while the value of ECC is <0.1, as shown in Figure 14d. On the other hand, for DHM, the S-parameters are moved to reduced frequency values, while maintaining the required operational bandwidth, as seen in Figure 15a, and the isolation among radiated components is >12 dB, as shown in Figure 15b. DHM’s efficiency has dropped to less than 50%, as illustrated in Figure 15c, and for Ant 1, the efficiency is at 35% since Ant 1 is so near to the palm. Moreover, the ECC value is smaller than 0.1, which is needed for MIMO operations, as shown in Figure 15d.

A thorough examination of the proposed work in relation to the current literature is undertaken and reported in Table 2. It is noted from the table that the suggested MIMO antenna has increased antenna efficiency compared to the designs presented in [5,6,8,9,10], and offers high CC compared to the MIMO antennas reported in [8,9]. Based on the performance attributes, it is believed that the proposed MIMO antenna system is a suitable candidate for sub-6 GHz 5G mobile communication systems.

## 6. Conclusions

In this study, an eight-element MIMO antenna is designed and described for sub-6 GHz 5G technology. A single antenna component is made of an L-shaped main element, an L-shaped parasitic element, and a rectangular slot in the ground plane. This enables the system to radiate at two different frequency ranges: 3.5 GHz and 4.8 GHz. The corner and the middle elements are arranged in an orthogonal manner that ensures spatial and pattern diversity with good isolation of at least 12 dB between any two given radiating elements. Various MIMO key performance parameters, such as ECC, MEG, and CC, are evaluated. It is observed that all the performance parameters comply with the requirements of MIMO systems. Furthermore, the gain of the proposed MIMO antenna is >2 dBi, and the total efficiency is ≥55% for both frequency ranges. For validation of simulation results, a prototype is fabricated and tested, and it is found that the modeled and actual results are quite close. To further demonstrate the proposed work as a potential antenna system for a modern mobile terminal, single- and double-hand mode analyses are studied. It is noted that for both scenarios, the proposed MIMO antenna system performs well for the desired frequency bands.

## Figures and Tables

**Table 1:** Computed MEGs of 8-element MIMO antenna.

Frequency (GHz)	MEG 1	MEG 2	MEG 3	MEG 4	MEG 5	MEG 6	MEG 7	MEG 8
3.5	−2.98	−3.67	−2.98	−2.87	−3.89	−4.12	−3.75	−3.99
4.8	−4.1	−3.46	−3.1	−2.99	−2.89	−2.99	−3.42	−2.87

**Table 2:** Comparative analysis among previously presented and proposed MIMO antennas.

Ref.	Board Size	No. of	Frequency Band	Total Efficiency	Isolation	ECC	Peak CC
	(mm${}^{2}$)	Elements	(GHz)	(%)	(dB)		(bps/Hz)
[5]	150 × 80	10	3.5/5.5	42/64	>15	<0.1	48/51.4
[6]	150 × 80	8	3.4–3.6	40–62	>17	<0.05	40.8
[8]	150 × 75	8	2.5–3.6	45–60	>13	<0.16	34.54
[9]	124 × 74	8	3.3–3.6	40	>15	<0.15	35
[10]	150 × 75	8	3.2–4	40–60	>12	<0.05	−
[19]	150 × 80	8	3.4–3.6	−	>10	<0.1	43
[20]	150 × 75	8	3.4–4.4	65–80	>10	<0.2	−
This Work	150 × 75	8	3.5/4.8 GHz	55/72	>12	<0.08	41

## Data Availability

Not applicable.
